# A Rare Case of Longitudinally Extensive Transverse Myelitis Following Febrile Illness: A Case Report

**DOI:** 10.7759/cureus.56316

**Published:** 2024-03-17

**Authors:** Indika Wettasinghe, Shiran Puthra, Hemal A Sugathapala, Suresh Mendis

**Affiliations:** 1 Internal Medicine, Colombo South Teaching Hospital, Colombo, LKA

**Keywords:** longitudinally extensive transverse myelitis (letm), bilateral lower limb paralysis, infectious disease, neurology, febrile illness

## Abstract

Longitudinally extensive transverse myelitis (LETM) is a rapidly progressing demyelinating disease affecting the spinal cord over three or more vertebral segments. Most causes are idiopathic, while others include infections, autoimmune causes, central nervous system demyelinating diseases, and post vaccination. Here, we report a 37-year-old male who presented with a fever for six days with no source of infection and complained of pain and weakness in the bilateral lower limbs eight hours after admission. Though the neurological examination of the lower limbs was normal at that time, reduced power was detected 16 hours later, with loss of proprioception and sensation of pain with a sensory level at T4 vertebrae. Then, the patient became unable to vocalize, and the chest X-ray showed an elevated left hemidiaphragm. Thirty hours after admission, the patient went into type 2 respiratory arrest and was intubated. The magnetic resonance imaging (MRI) showed a longitudinally extensive transverse myelitis extending from the C2 vertebrae to the conus medullaris.

Febrile illness is common in the medical setting in Sri Lanka, but its association with LETM is unusual. Since LETM is very rare and is a rapidly progressive disease, a high degree of clinical suspicion is crucial for early diagnosis and the initiation of treatment. This case underscores the importance of early diagnosis, which would require timely MRI, and prompt treatment with intravenous (IV) glucocorticoids or plasma exchange to reduce morbidity and mortality.

## Introduction

Febrile illness is a common presentation in Sri Lanka with dengue fever, leptospirosis, and typhus being the most common. A vast majority of patients are successfully managed by physicians in district hospitals all over the country. Severe myalgia is very common in these illnesses with dengue fever being known as “bone-breaking fever.” Liver involvement is a common complication in dengue fever, while both liver and renal involvement commonly occur in leptospirosis [1-3]. Neurological involvement, however, has received little attention in the past but is now being increasingly recognized [4]. Encephalopathy is the most common neurological manifestation of dengue, whereas aseptic meningitis and meningoencephalitis are the common neurological manifestations of leptospirosis [2,4].

Transverse myelitis is a rare but recognized complication of these illnesses. However, longitudinally extensive transverse myelitis (LETM) is extremely rare and has hardly been reported in Sri Lanka.

LETM is a rapidly progressing demyelinating disease affecting the spinal cord over three or more vertebral segments [5-8]. Most causes are idiopathic, while others include acquired central nervous system demyelinating disease (multiple sclerosis and neuromyelitis optica {NMO}), systemic autoimmune causes, infections, and post vaccination. Systemic autoimmune causes include antiphospholipid antibody syndrome, Behçet disease, mixed connective tissue disorder, neurosarcoidosis, Sjögren syndrome, and systemic lupus erythematosus [7-10].

Infective causes include viruses (adenovirus; coxsackie virus; cytomegalovirus, dengue virus; hepatitis A, B, C, and E virus; human immunodeficiency virus {HIV} type 1; etc.), bacteria (*Leptospira*, *Listeria*, *Mycobacterium tuberculosis*, *Treponema pallidum*, and *Borrelia burgdorferi*), parasites, and fungi [8]. *Burkholderia pseudomallei* is another important infective cause of LETM [11].

Here, we present a patient with a febrile illness, who was initially managed as leptospirosis on clinical judgement and developed an unusual complication of longitudinally extensive transverse myelitis.

## Case presentation

A 37-year-old male presented with a fever for six days. The symptoms were most severe on the first two days of illness, with high fever, arthralgia, myalgia, and headache. After day 2 of illness, the symptoms gradually improved, and the patient had only intermittent fever. On the day of admission, the patient had three episodes of vomiting, and he was catheterized at the local hospital since he had not passed urine the entire day.

There were no respiratory symptoms. There was no history of dysuria, and bowel opening was normal. He endorsed a history of a rat bite five days before the onset of fever. The tetanus toxoid but not the rabies vaccine was given on this day. There was no significant past medical, surgical, or allergy history. However, there was a significant history of leptospirosis exposure. He was a father of two with no history of intravenous (IV) drug abuse or sexual promiscuity.

On examination, the patient was febrile and icteric but not pale. There were no rashes nor eschar to be seen. The blood pressure was 110/70 mmHg, and the pulse rate was 100 beats per minute. The lungs were clear, and the abdomen was soft and nontender with normal genitalia. Three hundred milliliters of dark-colored urine was noted in the catheter. The neurological examination of the upper limb and lower limb was normal. A clinical diagnosis of leptospirosis was made, which was supported by the low platelets and deranged liver enzymes (Table 1).

**Table 1:** The biochemical parameters of the patient during hospital stay. WBC, white blood cells; RBC, red blood cells; HCT, hematocrit; MCV, mean corpuscular volume; MCHC, mean corpuscular hemoglobin concentration; AST, aspartate aminotransferase; ALT, alanine aminotransferase; RDW-SD, red cell distribution width-standard deviation; RDW-CV, red cell distribution width-coefficient of variation; GT, glutamyl transferase; CRP, C-reactive protein; ESR, erythrocyte sedimentation rate

Test	Normal Range	14/08/2022	15/08/2022	16/08/2022	20/08/2022
Full Blood Count					
WBC (×10^9^)	4.0-10.0	8.27	7.51	10.47	8.24
Neutrophils (×10^9^)	2.0-7.0	5.07	5.45	8.52	7.06
Lymphocytes (×10^9^)	1.0-3.0	2.66	1.55	0.92	0.61
Neutrophils (%)	50-70	61.3	72.5	81.4	85.7
Lymphocytes (%)	20-40	32.2	20.7	8.8	7.4
Monocytes (×10^9^)	0.2-1.0	0.45	-	0.9	-
Eosinophils (×10^9^)	0.02-0.5	0.04	-	0.0	-
Basophils (×10^9^)	0.02-0.1	0.05	-	0.03	-
Monocytes (%)	3.0-12.0	5.4	-	9.5	-
Eosinophils (%)	0.5-5.0	0.5	-	0	-
Basophils (%)	0.0-1.0	0.6	-	0.3	-
RBC (×10^12^)	4.5-5.5	3.97	-	4.07	-
Hemoglobin (g/dL)	12.0-16.0	13.3	12.3	13.2	10.4
HCT (%)	37-54	37.9	37.1	36.1	30.7
MCV (fL)	83-101	95.6	-	88.7	95.3
MCH (pg)	27-32	33.5	-	32.4	32.3
MCHC (g/dL)	31-34	35.1	-	36.6	33.9
RDW-SD (fL)	11.6-14.0	46.7	-	42.3	-
RDW-CV (%)	39.0-46.0	13.4	-	13	-
Platelets (×10^9^)	150-400	99	120	167	303
Renal Function Tests					
Sodium (mmol/L)	136-146	-	131.4	124	136
Potassium (mmol/L)	3.5-5.1	-	3.1	5.4	4.7
Urea (mmol/L)	2.8-7.2	-	5.3	-	-
Creatinine (µmol/L)	74-110	-	89.4	-	-
Calcium (mmol/L)	2.02-2.6	-	2.25	-	-
Albumin (g/L)	35-52	-	36.2	-	-
Corrected Calcium (mmol/L)	2.062-2.60	-	2.35	-	-
Phosphate (mg/dL)	2.9-5.0	-	-	-	-
Uric Acid (µmol/L)	210-420	-	340	-	-
Magnesium (mmol/L)	0.85-1.10	-	0.82	0.93	-
Liver Enzymes and Liver Function Tests					
AST (U/L)	<50	308	131	112	70
ALT (U/L)	<50	551	410	337	122
Total Protein (g/L)	66-83	68.1	66.85	-	-
Albumin (g/L)	35-52	36.2	30.9	-	41.8
Globulin (g/L)	25-35	34.4	35.9	-	-
Total Bilirubin (µmol/L)	5.0-21	242.8	160	-	37.6
Direct Bilirubin (µmol/L)	0.0-3.4	132.5	117	-	-
Indirect Bilirubin (µmol/L)	-	110.2	62.9	-	-
Gamma-GT (U/L)	<55	-	234.7	-	-
Inflammatory Markers					
CRP	<5	22	-	16	-
ESR	0-15	70	-	-	-
Procalcitonin	<0.07	-	-	2.01	-

Eight hours after admission, the patient complained of pain and weakness in the bilateral lower limbs. However, the muscle tone, power, and reflexes of the lower limbs were normal. The sensory examination of the lower limbs was normal, and the plantar was down-going. There was no tenderness along the spine.

Lower limb weakness was first detected 24 hours after admission. The muscle tone was normal, but the power was reduced to 3/5 in all movements of the hip and knee joints. The lower limb reflexes were normal, and the plantar was down-going. Sensory loss to pain was detected with a sensory level at the fourth thoracic vertebrae, and there was a loss of proprioception. The anal tone was normal.

**Figure 1:**
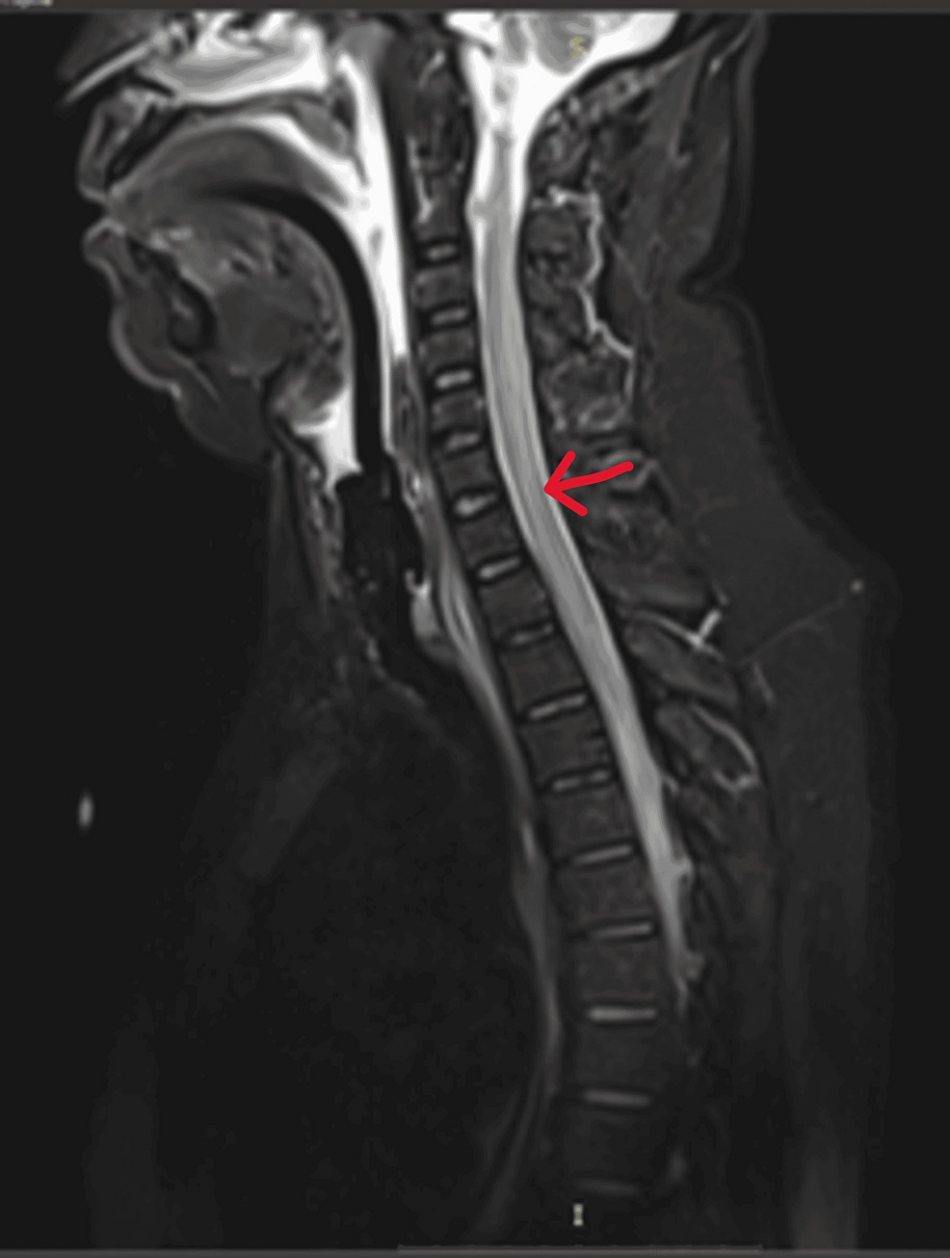
T2-weighted MRI of the cervical and thoracic spine (sagittal section) showing long segment intramedullary high signal intensities. MRI: magnetic resonance imaging

**Figure 2:**
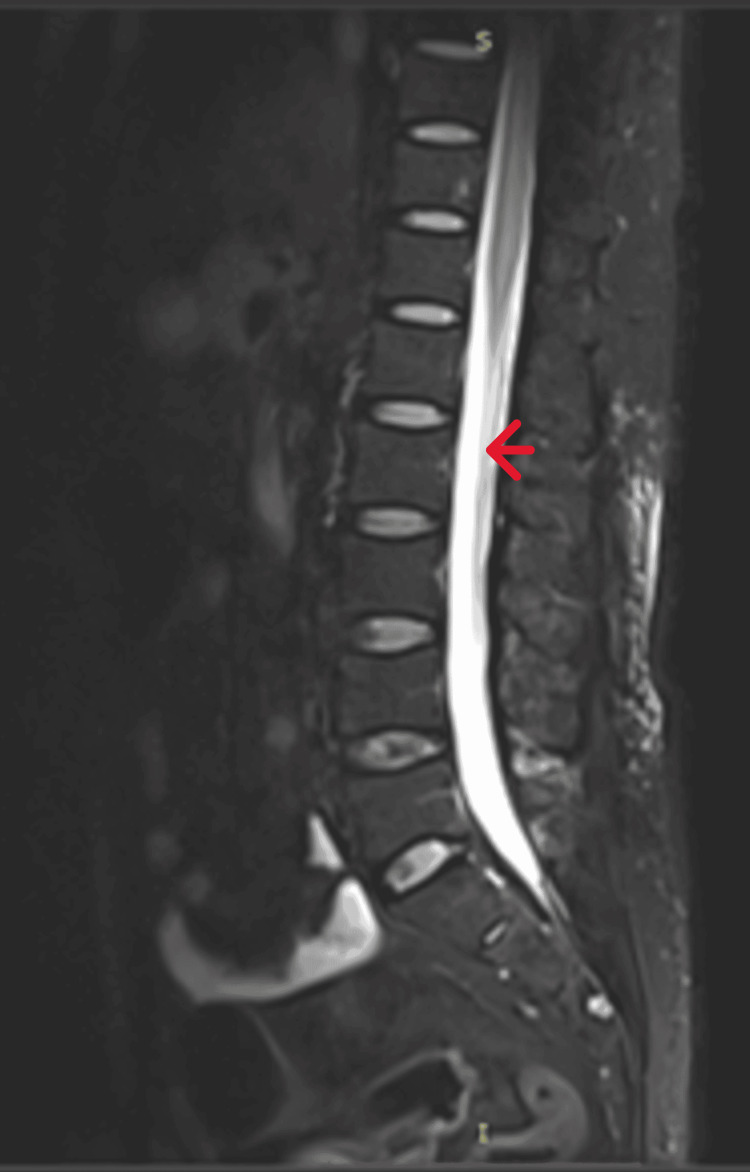
T2-weighted MRI of the lower thoracic, lumber, and sacral spine (sagittal section) showing long segment intramedullary high signal intensities extending up to the conus medullaris. MRI: magnetic resonance imaging

At this stage, it was decided to transfer the patient to a hospital with magnetic resonance imaging (MRI) facilities. However, before transfer (26 hours after admission), the patient became unable to vocalize (aphonia). There was no aphasia. The cranial nerve examination revealed that the uvula deviated to the left, while the rest of the cranial nerve examination was normal. The muscle power of the lower limbs was reduced to 2/5 for hip, knee, and ankle movements. The sensory loss of the lower limbs was unchanged, but the reflexes were still normal, and the plantar was down-going. The patient was transferred to the intensive care unit (ICU), and the chest X-ray (posterior-anterior {PA} view) taken on the way to the ICU showed an elevated left hemidiaphragm. The patient went into type 2 respiratory arrest in the ICU (30 hours after admission) and was intubated. He was then transferred for an MRI. The MRI showed long segment intramedullary high signal intensities extending from the level of C2 vertebrae to the conus medullaris. The lesion involved most of the cross-sectional area. Contrast images did not show an enhancement of the cord. However, there was cauda equina root enhancement without root thickening (Figures 1, 2).

The patient was treated with IV methylprednisolone 1 g daily for five days, followed by plasma exchange every other day for five days. Unfortunately, despite treatment, he did not recover, and he passed away on day 20 post admission from ventilator-associated pneumonia.

## Discussion

On presentation, the initial diagnoses for fever with icterus were leptospirosis, dengue fever, acute viral hepatitis, and typhus. Dengue and leptospirosis are well known to cause severe myalgia with the former being called bone-breaking fever as a result. Thus, the patient’s complaint of lower limb pain did not raise any alarms. However, with the onset of weakness, Guillain-Barre syndrome and transverse myelitis had to be considered with the infective causes being the most probable causative agents. The low urine output was thought to be due to acute kidney injury, but on retrospective analysis, it was due to urinary retention and would have provided the first clue of what was to come. In the patient records from the local hospital, there was no mention of a palpable bladder or of the urine output immediately after catheterization, which would have helped differentiate between urinary retention and acute kidney injury.

Once the clinical syndrome of myelopathy is recognized, an MRI with gadolinium enhancement is warranted [5]. Unfortunately, the neurological symptoms evolved rapidly within two hours, and the development of aphonia and right-sided phrenic nerve palsy forced a delay in doing the MRI as the patient was deemed not fit for transfer to a hospital with MRI facilities and was transferred to the ICU. It was only once that the patient was intubated following type 2 respiratory failure; then, the patient was transferred for an MRI.

The results of the investigations ordered to find an etiology are given in Tables 1, 2. The cerebrospinal fluid (CSF) analysis showed an albuminocytological dissociation with normal CSF glucose levels. Dengue IgG and IgM, *Leptospira* polymerase chain reaction (PCR), hepatitis B surface antigen, hepatitis C antibodies, venereal disease research laboratory (VDRL) test, and HIV antibodies were negative. Neuromyelitis optica (NMO) was excluded by fundoscopy and ophthalmology consultation. Investigations that we were not ordered due to limited resources, but should be considered in the ideal setting, are CSF oligoclonal bands, serum NMO-IgG, and myelin oligodendrocyte glycoprotein (MOG) antibodies to evaluate for neuromyelitis optica spectrum disorder and CSF viral studies (varicella zoster, *Enterovirus*, etc.), paraneoplastic panel, serum, and CSF angiotensin-converting enzyme to evaluate for sarcoidosis.

**Table 2:** CSF report and other serum investigations. CSF, cerebrospinal fluid; PCR, polymerase chain reaction; VDRL, venereal disease research laboratory; HIV, human immunodeficiency virus; EBV VCA, Epstein-Barr virus viral capsid antigen; ANA, antinuclear antibody

CSF Test	Result
Color	Colorless
Macroscopy	Clear
CSF Glucose	90.18
Protein (mg/dL)	111.1
Red Cells	22
Lymphocytes	1
Polymorphs	Nil
CSF Culture	Negative
Tuberculosis Gene Expert	Negative
Aquaporin 4 IgG	Not Available
Serum	
Dengue IgM and IgG	Negative
Leptospirosis PCR	Negative
Malaria	Negative
VDRL	Negative
HIV	Negative
Melioidosis Antibodies	Negative
Hepatitis B Surface Antigen	Negative
Hepatitis C Antibody	Negative
EBV VCA IgM Antibody	Nonreactive
Autoimmune	
ANA	Negative

Intravenous (IV) glucocorticoids are considered the first-line therapy in acute idiopathic transverse myelitis and should be initiated as soon as possible. IV methylprednisolone 1 g daily for 3-7 days is recommended [12,13].

Rescue therapy with plasma exchange may benefit patients who do not have a response to corticosteroids [14]. The recommended regimen is to exchange 1.1-1.5 plasma volumes, every other day, for 10 days (a total of five treatments). Another regimen is where the first two plasma exchange treatments are given on successive days, with the remaining three treatments given every other day [15].

This case highlights the significance of early diagnosis, which would require timely MRI and prompt treatment with IV glucocorticoids or plasma exchange. In regions lacking MRI facilities, clinicians should consider starting treatment if they are confident of the clinical diagnosis. To achieve this, they must maintain a high suspicion of LETM.

## Conclusions

Febrile illness is common in the medical setting in Sri Lanka with most patients complaining of myalgia. However, this case shows an unexpected association with LETM. Since LETM is very rare and is a rapidly progressive disease, a high degree of clinical suspicion is crucial for early diagnosis and the initiation of treatment. Urgent MRI for diagnosis and prompt treatment with IV glucocorticoids or plasma exchange is essential to prevent the rapid progression of the disease and thereby reduce morbidity and mortality.
